# The Dynamic Interactions between *Salmonella* and the Microbiota, within the Challenging Niche of the Gastrointestinal Tract

**DOI:** 10.1155/2014/846049

**Published:** 2014-07-10

**Authors:** C. M. Anjam Khan

**Affiliations:** Centre for Bacterial Cell Biology, Institute for Cell and Molecular Biosciences, The Medical School, University of Newcastle, England NE2 4HH, UK

## Abstract

Understanding how *Salmonella* species establish successful infections remains a foremost research priority. This gastrointestinal pathogen not only faces the hostile defenses of the host's immune system, but also faces fierce competition from the large and diverse community of microbiota for space and nutrients. *Salmonella* have solved these challenges ingeniously. To jump-start growth, *Salmonella* steal hydrogen produced by the gastrointestinal microbiota. Type 3 effector proteins are subsequently secreted by *Salmonella* to trigger potent inflammatory responses, which generate the alternative terminal electron acceptors tetrathionate and nitrate. *Salmonella* exclusively utilize these electron acceptors for anaerobic respiration, permitting metabolic access to abundant substrates such as ethanolamine to power growth blooms. Chemotaxis and flagella-mediated motility enable the identification of nutritionally beneficial niches. The resulting growth blooms also promote horizontal gene transfer amongst the resident microbes. Within the gastrointestinal tract there are opportunities for chemical signaling between host cells, the microbiota, and *Salmonella*. Host produced catecholamines and bacterial autoinducers form components of this chemical dialogue leading to dynamic interactions. Thus, *Salmonella* have developed remarkable strategies to initially shield against host defenses and to transiently compete against the intestinal microbiota leading to successful infections. However, the immunocompetent host is subsequently able to reestablish control and clear the infection.

## 1. Introduction

*Salmonella* is a major pathogen of the gastrointestinal tract. Our understanding of the cellular and molecular mechanisms by which* Salmonella *causes disease has been greatly advanced in recent years. In parallel, over the past decade there have been remarkable insights made into our understanding of the human microbiota and its importance in health and disease. Much of this work has largely been descriptive but it has provided essential knowledge on the composition of these communities. For example, the composition of the intestinal microbiota has been determined and provided insightful correlations to health and a variety of disease states, including infections caused by bacterial pathogens. Recently there have been surprising revelations on the dynamic interactions between* Salmonella* and the intestinal microbiota, as they both compete for essential nutrients and space. The gastrointestinal tract provides a rich and hospitable environment for the microbiota, but the host defends this territory fiercely against invading bacterial pathogens. Remarkably, the hosts frontline defense system is exploited by* Salmonella*, to inadvertently fuel the “battle of the bugs” and promote a transient growth bloom of* Salmonella* at the expense of the microbiota and also in detriment to the host. Ultimately, the host is able to successfully reestablish control and clear the infection. This review will provide an introduction on our knowledge and understanding of the biology and pathogenicity of* Salmonella* and subsequently focus on some of the latest research developments that have provided vivid insights into the dynamic interactions between* S. Typhimurium*, the resident intestinal microbiota, and the gastrointestinal tract.

## 2. *Salmonella*: A Model Enteric Pathogen

The biology and virulence of* Salmonella* have been intensively studied, providing vivid insights into the mechanisms by which this ingenious pathogen causes disease in mammalian hosts. Key aspects of the virulence mechanisms are discussed below in the context of the biology of* Salmonella*.

### 2.1. *Salmonella* Are Major Global Pathogens

Salmonellae are Gram-negative facultative intracellular bacterial pathogens, which can infect a broad range of animals [1, 2].* Salmonella* are rod-shaped bacteria varying in size from about 2 to 3 *μ*m by 0.4 to 0.6 *μ*m, and their rod shape is maintained by an actin-like bacterial cytoskeleton [3, 4].* Salmonella* species cause an extensive spectrum of disease in humans from mild gastroenteritis to life-threatening Typhoid fever [5]. Salmonellosis remains a major global health problem causing significant morbidity and mortality. Annually there are 16 million cases of Typhoid fever, 1 billion cases of gastroenteritis, and 3 million deaths across the globe.

Most species can infect a broad range of hosts as is the case for* Salmonella enterica* serovar Typhimurium (*S. Typhimurium*), but other serotypes such as* S.* Typhi,* S. Pullorum*, and* S. Gallinarum* are exquisitely host-restricted [6]. Infections in humans lead to acute gastroenteritis, manifested with the clinical symptoms of abdominal pains, nausea, diarrhea, and vomiting. The organisms most frequently associated with this diarrheal disease are* S. Typhimurium* and* S. enterica* serovar Enteritidis (*S. Enteritidis*) and are the leading agents responsible for acute gastroenteritis [7]. In healthy individuals salmonellosis is a self-limiting infection but in the young, the elderly, or the immunosuppressed,* Salmonella* infections can lead to severe complications and possibly death. In such individuals the infection can spread systemically following breach of the gastrointestinal tract and internalization within phagocytes [8]. Cephalosporins and fluoroquinolones are the preferred antibiotics for treatment of these patients [9].

*Salmonella Species Are Antigenically and Genetically Diverse*. The* Salmonella* genus is divided into two species* Salmonella bongori* and* Salmonella enterica*. The species* Salmonella enterica* is divided further into six subspecies. Based on the presence of distinct surface antigens salmonellae can be divided into over 2500 serovars based on the scheme established by Kauffman and White a century ago [10]. These include flagellar H antigens, somatic lipopolysaccharide O antigens, and virulence (Vi capsular K antigens [10]. From these six subspecies,* Salmonella enterica* subspecies* enterica* represents 60% of the 2500 serovars [10], is most often linked with disease, and is the most diverse. Using whole genome sequence analysis,* S. enterica* subspecies* enterica* could be subdivided into two populations, namely, clade A and clade B, reflecting differences in host and tissue tropisms and metabolism [11].

Achtman and colleagues have also recently used an alternative DNA sequence based approach to investigate by serology [12]. Using multilocus sequence-based typing (MLST) to identify evolutionary relationships, the authors designated the term eBurst groups (eBGs) to signify closely related genetic clusters. Some eBGs matched serovars but many of the clusters identified surprisingly contained multiple serovars. These results clearly suggest that serovar classifications may misdirect epidemiologists and clinicians investigating* Salmonella* outbreaks and that a DNA-based approach for classification is much needed [12].

The* Escherichia coli* (*E. coli*) and* Salmonella enterica* (*S. enterica*) diverged approximately 100 million years ago and their genomes differ by 10%. The* S. enterica* serovars core genomes differ by less than 1% with each other. They are just under 5 Mb in size and encode for approximately 4,500 genes [11, 13, 14]. The genomes of the enterics have been under intense selective pressures and this is evident in their mosaic genomes.* Salmonella* have acquired blocks of DNA referred to as* Salmonella* pathogenicity islands (SPIs) as they encode genes important for virulence [15]. The GC content of this DNA differs from the core genome and the pathogenicity island appears to be integrated into redundant tRNA genes [16]. This suggests they may have been derived from a different bacterial species. Pathogenicity islands provide “quantum leaps” in evolution of bacteria by providing preassembled packages of virulence genes [17]. There are approximately 20 SPIs in* Salmonella* distributed around the circular chromosome [18]. In addition there is further genetic diversity provided by plasmids, islets, prophages, and phage remnants. Superimposed on top of this variation,* Salmonella* can undergo genomic rearrangements involving recombination between different rRNA operons and insertion sequences [19, 20].

*Chronic Asymptomatic Carriage.* In some individuals infection with* S. *Typhi can lead to chronic asymptomatic carriage [21]. These individuals shed large numbers of bacteria in their feces and can subsequently transmit the pathogen to naïve hosts by contamination of food and water sources. The most famous carrier case is Mary Malone, who was a cook in New York in the early 1900s and became known as “Typhoid Mary” as she transmitted the infection to many others. Livestock have also been identified as carriers, and shedding may play a vital role in epidemics [22, 23].

*Emergence and Spread of Multidrug Resistant and Hyper-Virulent Strains.* The health and economic burden of this disease is likely to increase due to the rise in multidrug resistant strains and the emergence of new “hyper-virulent” variants [24]. Dougan and colleagues have investigated the emergence in sub-Saharan Africa of a highly invasive nontyphoidal* Salmonella* (iNTS) strain ST313, a variant of* S. Typhimurium* [24]. Rather than remaining largely localized around the intestines this variant can now breach the intestinal barrier and colonize humans systemically. Furthermore, the strain appears to have spread from human to human rather than as a zoonosis. Using the power of whole genome sequencing, the investigators observed two distinct lineages of iNTS that appeared approximately 52 to 35 years earlier, correlating in time with the HIV epidemic and treatment of the iNTS with chloramphenicol. Furthermore the strains had accumulated pseudogenes, rather like the invasive and host-restricted* S.* Typhi responsible for Typhoid fever. These pseudogenes may have promoted the systemic spread of the strains and disease [24, 25]. Thus, immunodeficiency and widespread antibiotic use have been powerful selective forces in the emergence of these highly virulent and drug-resistant strains [24].

The multidrug resistant* S. Typhimurium* DT104 has been responsible for global epidemics during the 1990s [26]. DT104 was found to be resistant to ampicillin, chloramphenicol, streptomycin, sulphonamides, and tetracycline and the resistance type is referred to with the acronym ACSSuT [26]. The genes encoding the antibiotic resistance were found to be chromosomally encoded within a locus termed* Salmonella* genomic island (SGI-1), which is a 43 kb integron [27]. It was popularly believed that this DT104 epidemic was largely a zoonotic infection acquired from eating food products derived from cattle. Using a unique collection of 200 DT104 isolates from a limited geographical area of Scotland, obtained over a 20-year timeframe, Nick Thomson and colleagues began their molecular detective work [28]. Using whole genome sequencing, they investigated the associations between the strains and antibiotic resistance genes [28]. The phylogenetic relationships were established using whole genome sequencing. The results were epidemiologically surprising and provided overwhelming evidence to suggest that contrary to popular belief, the* S. Typhimurium* DT104 epidemic strains and antibiotic resistance genes were maintained largely independently within human and food animal epidemics, with limited exchange. Antibiotic resistance genes can be transferred horizontally to recipient bacteria as will be discussed in a later section. The authors observed an equally high variability in antibiotic resistance profiles between human and animal isolates. These findings highlight the importance of collecting genotypic data to facilitate our understanding of the ecological basis of epidemics and thus enabling the correct implementation of targeted control measures [28].

*Colonization Beyond Animals.* There is an increasing body of evidence to suggest that* Salmonella* can infect nonanimal hosts and colonize agriculturally important plants [29]. In fact many* Salmonella* serovars can attach to plants more strongly than the infamous* Escherichia coli* 0157:H7 [30]. Following attachment,* Salmonella* can colonize internal plant tissues using virulence genes, which also play an important role in the colonization of animal hosts [31]. Transmission of* Salmonella* from raw fruit and vegetables to humans is now being increasingly recognized as a major proportion of the reported cases of food poisoning in the United States [32].

### 2.2. The Infectious Cycle of* Salmonella*

*The Intestinal Phase*. Infections with* Salmonella* are normally acquired by ingestion of contaminated food and water. Once ingested* Salmonella* encounter the acidic pH of the stomach which induces an acid tolerance survival response, leading to physiological changes and enabling survival of a small subpopulation [33]. Upon entry into the small intestine,* Salmonella* are exposed to the destructive effects of the luminal contents including bile salts, enzymes, antimicrobial peptides, and secretory IgA [34, 35].* Salmonella* have at least four different infection pathways, namely, via, M-cells, intestinal epithelial cells, CD18-expressing phagocytes, or across a damaged intestinal barrier.

*Salmonella* travel through the mucous layer to invade intestinal epithelial cells, M-cells, and dendritic cells (DCs). Intestinal epithelial cells are normally nonphagocytic; however,* Salmonella* have the capacity to invade these cells through a sophisticated machinery termed a type 3 secretion system (T3SS); the genes encoding the T3SS are located on* Salmonella *pathogenicity island 1 (SPI-1) [36, 37].* Salmonella* target specialized M-cells and this initial attachment is promoted by fimbrae [38]. M-cells sit on top of the lymphoid follicles of Peyer's patches which are normally involved in the pinocytotic sampling of luminal antigens, and this process plays a key role in mucosal immunity. During the process of invasion,* Salmonella *induce membrane ruffles, which surround the pathogen leading to endocytosis [39]. M-cells transport* Salmonella* across the epithelial barrier to DCs, which play a key role in innate immune responses.

However, following phagocytosis,* Salmonella* are able to survive and manipulate the function of the host cell by using effectors of the SPI-2 T3SS (see below), to interfere with, for example, antigen processing and presentation [40].* Salmonella* are now able to spread systemically by using DCs as vehicles.* Salmonella* can also use an alternative strategy to disseminate from the gastrointestinal tract by being taken up by CD18-expressing phagocytes [41]. These phagocytes can transmigrate across tissue barriers and transport* Salmonella* from the lumen of the gastrointestinal tract to the systemic circulation.* Salmonella* can also disrupt tight junctions between adjacent epithelial cells, which normally help prevent leakage of water, ions, nutrients, and immune cells from the gastrointestinal tract [42].

*The Systemic Phase.* Once* Salmonella *break across the epithelial cell barrier they are taken up by intestinal macrophages and can initiate systemic disease. In the hostile environment of the phagosome,* Salmonella* turn on the expression of genes in another horizontally acquired pathogenicity island termed SPI-2 T3SS [43, 44]. The resulting gene products modify the phagosome into a* Salmonella* containing vacuole (SCV) and remodel the host cell [45]. Collectively these changes lead to a more hospitable environment for the survival and replication of* Salmonella*. The pathogen can now become disseminated throughout the reticuloendothelial system. In most healthy individuals,* Salmonella* remain localized to the gastrointestinal tract, which leads to a major migration of neutrophils into intestinal lumen and results in an inflammatory response leading to diarrhea [46]. In most patients the intestinal infection is limited to the small intestine, but in a distinct proportion it involves the colon (colitis). There is a major neutrophil influx into intestinal tissues during human enterocolitis [46, 47]. Stool samples of these enterocolitis patients contain leukocytes dominated by the presence of neutrophils. In otherwise healthy patients,* S. Typhimurium* infection is self-limiting leading to clearance typically within 14 days and does not require antibiotic treatment [48].

### 2.3. *Salmonella's* Virulence Machinery Promotes Survival and Transmission

*Salmonella enterica* encode a variety of virulence factors to promote survival, replication, and transmission of the pathogen into new hosts [49, 50]. Of particular importance to virulence are the type 3 secretion systems (T3SSs) [51]. These secretion systems enable the pathogen to directly inject bacterial effector proteins through a molecular needle-like structure into a host cell to subvert its function.* Salmonella* harbor two T3SSs encoded on pathogenicity islands SPI-1 and SPI-2. Through the innovative work of Jorge Galan and David Holden and colleagues respectively, vivid insights have been provided into the importance and workings of these two elegant systems [37, 52].

*The SPI-1 Type 3 Secretion System Is Important for the Intestinal Phase of Infections.* The SPI-1 system appears to be important for the intestinal phase of infection, whereas the SPI-2 system is essential for establishing the systemic phase. These generalized timings for the expression of SPI-1 and SPI-2 genes are now becoming less clearly demarcated [53, 54]. The T3SSs encode for 20 to 30 proteins involved in the assembly of the macromolecular structure termed the needle-complex. The proteins can be functionally classed as being structural, regulatory, chaperones, or effectors of virulence. The T3SS complex basal body spans both the inner and outer bacterial membranes and then continues with a narrower needle complex projecting out from the cell [55]. The SPI-1 T3SS basal body structure is composed of the proteins PrgH, PrgK, and InvG, and the needle itself is composed of polymers of a single subunit protein PrgI. This structure resembles flagella, suggesting an evolutionary connection between these organelles. This is further supported by the observation that T3S structural proteins share sequence identity with flagella proteins [56]. At least three additional proteins are required for the translocation of effector substrates in to host cells, namely, SipB, SipC, and SipD. The assembly of the complex structure takes place through a programmed series of distinct steps, with similarities again to the flagella assembly pathway. Once the structure is fully assembled and energized it is now competent to directly translocate effector substrates in to host cells from the bacterial cytoplasm, without a periplasmic or extracellular phase. There are over 13 effector proteins and a number of which are encoded outside of SPI-1, including SPI-5 and prophages. These proteins include AvrA, SipA, SipB, SipC, SipD, SlrP, SopA, SopB, SopD, SopE, SopE2, SptP, and SspH1, and the list is ever expanding [50, 51, 57]. Indeed SopE is encoded by a lysogenic bacteriophage [58]. As there is no direct evidence to support the notion that effector molecules are transported directly through the needle-complex, a radically alternative view has been suggested based on data with* Yersinia* [59]. This model, by Hans Wolf-Watz and colleagues, represents a radical shift in our thinking to suggest the needle-complex may in fact play a role in target cell sensing and that effector proteins are secreted through a pore in the host cell membrane [59]. Further, research is needed to validate these secretion models and also it must be noted that they are not mutually exclusive.

*Bacterial Mediated Endocytosis.* Key SPI-1 T3SS effector proteins are involved in the process of bacterial-mediated endocytosis, and these include SipA, SipC, SopB, SopE, and SopE2. In nonphagocytic cells such as intestinal epithelial cells, these effectors instigate membrane ruffling and engulfment of the bacteria by the “Trigger” mechanism [60, 61]. Koronakis and colleagues have observed that many effectors have acquired multiple functions. For example, SipC is involved in both actin bundling and also actin nucleation [60, 61]. SipA also induces actin bundling and polymerization and formation of the SCV. Furthermore, SipA stimulates neutrophil transmigration and remarkably processes and activates caspase-3 [62, 63].

*Receptor-Mediated “Zipper” Entry.* A variety of independent experimental approaches have recently provided evidence which challenges the above established “Trigger” mechanism paradigm and suggests that invasion of nonphagocytic host cells by* Salmonella* can also occur by the “Zipper” mechanism [64]. The* Salmonella* outer membrane proteins Rck and PagN appear to function as invasins and promote cell invasion. The entry mechanism is independent of T3SSs [65].

*Induction of Potent Inflammatory Responses.* Following internalization, the* Salmonella* effectors induce a potent inflammatory response. The effectors SopB, SopE, and SopE2 activate the small GTPases Cdc42 and Rac-1, stimulating the mitogen-activated protein kinase (MAPK) pathway leading to downstream activation of the transcription factors: activator protein 1 (AP1) and nuclear factor kappa-B (NF-*κ*B) [66]. AP1 and NF-*κ*B elicit the transcription of proinflammatory cytokines such as IL8, which stimulates the transmigration of neutrophils to the intestinal mucosa. SipA alone, as mentioned above, can also trigger signal cascades leading to the transmigration of neutrophils into the intestinal lumen [67]. In addition, SipB has the functionality to activate the caspase-1 inflammasome leading to increases in levels of the proinflammatory cytokines IL-1*β* and IL-18 [68]. It has also been observed that SopB, SopE, SopE2, and SipA can transiently disrupt epithelial cell tight-junctions and thus disrupt the intestinal barrier, enabling an influx of neutrophils. Through its inositol phosphatase activity SopB stimulates chloride ion secretion and ion fluxes [69, 70]. This leads to the characteristic inflammatory diarrhea, an important feature of gastroenteritis.

*The SPI-2 Type 3 Secretion System Is Essential for the Systemic Phase of Infection.* Whilst screening for genes essential for systemic growth of* Salmonella* in mice, Holden and colleagues discovered the SPI-2 T3SS using the innovative technique of signature-tagged mutagenesis [71, 72]. The SPI-2 T3S apparatus has a very similar structure to the SPI-1 T3S needle complex. When* Salmonella* are taken up or phagocytosed by cells, for example, macrophages, they develop within the* Salmonella* containing vacuole (SCV). The SCV possesses features of a late endosome including an acidic pH but lacks other characteristic endosome markers and is therefore considered a distinct entity [52]. It has been proposed that the main function of the SPI-2 T3SS is to modify host cell vesicular trafficking, enabling important metabolites to be routed to the SCV and thus providing a safe replication niche. The SPI-2 T3SS genes encoding the secretion machinery are regulated by two-component systems, which include OmpR-EnvZ and also SsrA-B located on SPI-2 itself. The regulatory systems sense the acidic pH and the weak nutritional environment within the SCV. Once a translocon pore is assembled in the SCV membrane, the host cell cytoplasmic pH is sensed, resulting in disassembly of the regulatory complex composed of SpiC, SsaL, and SsaM [73]. This triggers the derepression of delivery of around 30 effector proteins. The effector proteins SifA, SopD2, SseJ, and PipB2 function in the maintenance of the SCV membrane. The effectors SseF and SseG regulate the SCV's juxtanuclear position near the Golgi apparatus. Effectors also target the host cell cytoskeleton such as SteC, SpvB, SspH2, and SrfH. A number of effectors including SpvC, SspH1, and SseL play key roles in ubiquitin modification and host immune signaling [74].

## 3. The Microbiota Live in Intimate Associations with Human Hosts

Complex microbial communities live in intimate association with their human hosts in commensal and also pathogenic interactions. Our understanding of the human microbiota has been completely revolutionized by the pioneering research of Gordon and colleagues [75, 76]. The huge health benefits members of these microbial inhabitants can provide to their hosts are only just beginning to be fully realized and can impact upon diverse conditions as allergies, obesity, diabetes, cancer, and autism. In the sections below we will discuss the benefits the intestinal microbiota can provide against invading bacterial enteropathogens such as* Salmonella*.

### 3.1. The Microbiota Is a Large and Diverse Community

**Figure 1:**
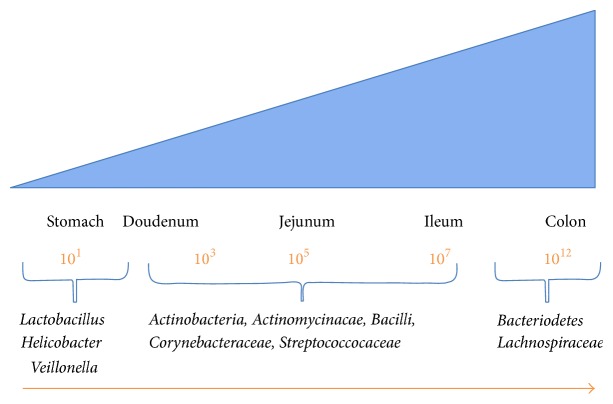
The microbiota population steadily increases along the length of the gastrointestinal tract from the stomach to the colon. The major resident bacterial phyla/species are indicated, together with the numbers of bacterial cells per gram of luminal contents.

A diverse spectrum of microbial communities live in intimate associations with their human hosts and are termed the microbiota [77]. These complex communities contain members from all three domains of life, namely, bacteria, archea, and eukarya. Trillions of bacteria colonize and reside on surfaces within and upon us in mainly a symbiotic relationship. The structure and complexity of these microbial ecosystems vary depending on their location, for example, from the skin to the mouth to the intestine. These microbial communities also provide important ecological benefits to their human hosts based on their locations. In healthy individuals there is a balanced relationship with the microbes, with no resulting disease [75]. The numerical importance of the microbiota can be gauged if we adopt a metrics system of cell numbers, in which case humans equate to being only 10% human and 90% microbe [78, 79]. The diverse population of bacteria that inhabit our bodies is collectively referred to as the bacterial microbiota, and for the purpose of this review we will refer to it simply as the microbiota hereafter. The resident microbiota is in a state of continual flux and undergoes changes from birth through to adulthood of their hosts. They play a key role in maintaining human health by a variety of mechanisms [80]. The human gastrointestinal tract has a large surface area of 200 m^2^ and the lumen is rich in nutrients [81, 82]. Consequently the gastrointestinal tract provides a very hospitable niche for bacteria. The gastrointestinal tract contains up to 100 trillion bacteria across approximately 1000 species in humans [82, 83]. However, only a small proportion of this microbiota can be cultured and the studies have been reliant on high-throughput sequencing methods to characterize the full range of species present in the microbiota. The resident microbiota colonizes the length and width of the gastrointestinal tract, steadily increasing in numbers from the stomach and duodenum. They vary from as few as 10^1^ bacteria per gram of luminal content in the stomach to 10^6^ bacteria per gram of luminal content in the jejunum and to as many as 10^12^ bacteria per gram of luminal content in the colon (Figure 1). The colonic bacteria are largely facultative anaerobes and are dominated by the phyla Bacteroidetes and Firmicutes [80]. Actinobacteria, Proteobacteria, Cyanobacteria, Verrucomicrobia, and Fusobacteria are present as a minor fraction of microbiota[84].

### 3.2. The Collective Gene Content of the Intestinal Microbiota Is Vast and Provides Huge Benefits for the Host

This enormous community of resident bacteria and their genomes collectively encode the gut “microbiome,” and the microbiome contains more than 100-fold greater number of genes than the human host. The microbiome encodes for biochemical pathways not present in humans which breakdown complex indigestible polysaccharides and proteins. These pathways release and generate essential metabolites including amino acids, vitamins K and B, and short chain fatty acids and are now metabolically available to the benefit of the host. These resident bacteria therefore provide huge paybacks to their hosts. They appear to play key roles promoting the development of the immune system and promoting health. The microbiota can also afford the host protection against invading bacterial pathogens in a process known as “colonization resistance” and will be discussed later [85].

*We Are What We Eat: Diet Shapes the Composition of the Gastrointestinal Microbiota. *Diet has a pivotal impact on shaping the composition of the gastrointestinal microbiota [86]. By analyzing detailed nutritional intake in 98 adults with the composition of the microbiota using 16S rDNA sequencing of fecal samples, Wu et al. observed that long-term diet shaped the microbiota enterotypes. For example, intake of animal fat and protein versus carbohydrates correlated with an increased association with* Bacteroides* or* Prevotella* species, respectively [86]. Based upon these observations, Turnbaugh and colleagues investigated the short-term impacts of diet changes on the plasticity of the gastrointestinal microbiota using 16S rDNA sequencing [87]. They studied the effects of dietary regimes based on either animal or plant products and found that surprisingly even within a single day, the bacterial community rapidly changed in response to diet [87]. The animal-based diet led to an increase in bacteria tolerant to bile such as* Alistipes*,* Bilophila*, and* Bacteroides*. Furthermore, there were reductions in the numbers of Firmicutes, which metabolize dietary plant polysaccharides including* Roseburia*,* Ruminococcus bromii*, and* Eubacterium rectale* [87]. The bacterial gene expression profiles of specific metabolic modules and pathways also became altered as assessed by RNA sequencing (RNA-seq).

*Temporal Stability of the Gastrointestinal Microbiota. *There has been a major gap in our knowledge on understanding of how the composition of the microbiota changes throughout the life of an individual. Another landmark study by Jeff Gordon and colleagues has recently addressed this issue by examining fecal microbiota in 37 adults [88]. They determined the composition of the microbiota from these individuals by developing a quantitative low-error 16S ribosomal RNA amplicon sequencing (LEA-seq) method and used this in conjunction with whole-genome sequencing. They observed that 60% of the strains or phylotypes remained stable for 5 years [88]. This stability followed a power-law function and could be statistically extrapolated to potentially remaining stable for decades. In particular Bacteroidetes and Actinobacteria are significantly more stable components than other members of the microbiota. Faith et al. observed that early gastrointestinal tract colonizing microbiota are acquired from our close family members and can potentially mediate their biological effects on health for a significant part of their lives [88].

*Antibiotics Impact on the Composition of the Microbiota.* The stability in the composition of the microbiota communities in humans can be modulated by a variety of factors including disease, diet, and medicines. For example, the use of antibiotics in treating infections in humans has a striking effect on the microbiota with a reduction in various taxa and a consequent decrease in bacterial diversity [89]. David Relman and colleagues investigated the effects of the antibiotic ciprofloxacin on the microbiota in three healthy individuals using deep 16S RNA sequencing. Approximately one-third of the bacterial taxa in the gut were reduced in population following antibiotic treatment, and this effect varied amongst individuals. Furthermore, within 4 weeks the composition of the community recovered to preantibiotic treatment levels in all three adults. However, some taxa did not recover even within 6 months [89]. Thus, antibiotic treatments can impact upon the diversity of the microbiota community and as a consequence potentially have longer term impacts on health and disease in hosts.

*Postantibiotic Expansion of Enteric Pathogens.* The administration of oral antibiotics to humans clearly disrupts the intestinal microbiota and increases the risks associated with acquiring* Salmonella* and* Clostridium difficile *infections. A very recent study by Sonnenburg and colleagues provided remarkable mechanistic insights into these observations [90]. These authors used gnotobiotic mice, mono-associated with the* Bacteroides thetaiotaomicron* and subsequently infected with* S. Typhimurium*. Following recovery of the pathogen from caecum contents it was subjected to transcriptional profiling. This analysis revealed that genes involved in carbohydrate metabolism were highly upregulated, including three operons involved in the catabolism of sialic acid, fucose, and propanediol, a catabolite of fucose [90].* S. Typhimurium* mutants were constructed defective for utilization of sialic acid and fucose and were found to be significantly less competitive than the parent strain in mixed infections in vivo. Compared to germ-free mice,* B. thetaiotaomicron* colonized mice contained much greater levels of free sialic acid (*N*-acetylneuraminic acid).* B. thetaiotaomicron* possesses the enzyme sialidase, which cleaves the terminal sugar from mucosal conjugates. However, if gnotobiotic mice were colonized by a sialidase deficient* B. thetaiotaomicron* mutant, this significantly reduced pathogen expansion following infection. Sialic acid concentrations were next determined in the ceca of conventional mice following oral antibiotic treatment and revealed a spike in concentration compared to untreated mice. However,* S. Typhimurium* mutants lacking the ability to catabolize sialic acid were unable to fuel their growth in the mouse intestinal tract following antibiotic treatment. Collectively these data suggest that disruption of the microbiota modulates carbohydrate availability, which is exploited by enteric bacterial pathogens to promote growth. These findings have important therapeutic implications [90].

### 3.3. Model Systems for Studying* Salmonella* Infections and Interactions with the Microbiota

The conventional mouse is the most common model system used to study* S. Typhimurium* infection and more recently the interactions with the microbiota. Mice can be resistant or genetically susceptible to* Salmonella* infection. Infection of BALB/c or C57BL/6 mice with* S. Typhimurium* resembles many aspects of the infection of humans by the host-restricted* S.* Typhi, leading to Typhoid fever. BALB/c or C57BL/6 mice have a mutation in the gene formerly known as* Nramp1* (natural resistance-associated macrophage protein one) and renamed the* Slc11a1* gene that leaves them susceptible to systemic infection by* S. Typhimurium*. The mutation results in a defect in the membrane-bound divalent cation, that is, the Fe^2+^ anti-porter within the* Salmonella *containing vacuole in macrophages [91]. The mouse system also offers considerable technical advantages in the availability of immunological tools and transgenic knockout mice.

The mouse model can be adapted to study gastroenteritis by disrupting the normal microbiota using antibiotics [92]. The most popular antibiotic for this purpose is streptomycin. Streptomycin-treated mice when infected with* S. Typhimurium* develop a disease, which resembles more closely human gastroenteritis, rather than systemic disease. These mice develop an exudative intestinal inflammation with migrated neutrophils in the caecum.

On the other hand, gnotobiotic mice contain defined intestinal microbiota within the gastrointestinal tract and are an attractive model system to study the impact of resident intestinal microbiota on the process of infection [93]. They can range from abiotic germ-free mice, which have no microbiota, to monoassociated or polyassociated mice colonized by known bacterial communities.

The mouse systems have provided excellent models for studying systemic disease. They have yielded a huge wealth of information on the biology and pathogenicity of* Salmonella* and its interactions with the host and microbiota. However, the mouse model is of very limited use in studying gastroenteritis. Thus, in contrast to mice, natural or experimental infection of calves with* S*.* Typhimurium* leads to a localized intestinal disease with many of the hallmarks of a human infection from clinical symptoms to pathology [94]. The calve model offers many practical and technical challenges, however, with only a limited set of genetic and immunological tools, as well as the ethical and financial issues involved.

### 3.4. The Microbiota Protect the Host from Pathogens by Providing Colonization Resistance

The gastrointestinal tract represents a vast mucosal surface area vulnerable to attack by enteropathogens. The gastrointestinal tract is fortified with a variety of physical and immunological defence barriers. A major protective shield for the gastrointestinal tract and only more recently recognised is the colonising microbiota. This dense population of microbiota is thought to provide both a physical barrier for the attachment of bacterial pathogens to the mucosal surfaces and outcompeting invading pathogens for essential nutrients. This protective mechanism has been termed “colonization resistance” and helps prevent invading bacteria under normal conditions establishing an infection [85].

The streptomycin-mouse model has provided an important system to study colonization resistance and colitis following* S. Typhimurium* infection. Oral treatment of mice with the antibiotic streptomycin reduces the microbiota population by approximately 80% and reduces the colonization resistance for window of 24 hours or so. If the mice are now infected with* S. Typhimurium* this leads now to very efficient colonization of the intestine and especially the cecum and colon where densities as high as 10^9^ colony forming units per gram have been reported [95]. The microbiota population takes around 36 hours to grow back to original levels.

The microbiota also occupies and blocks the binding of pathogens to attachment sites present in the mucous layer. These include carbohydrate groups present within the mucus layer. Many of the specific receptor-ligand interactions that take place still remain to be determined.* Bacteroides thetaiotaomicron* is an important colonizer of the mucus layer and digests mucin peptides and O-linked glycans as an energy source, resulting in the production of short chain fatty acids (SCFA) such as butyrate [96, 97]. The microbiota is able to produce a nutritional environment not favorable for the growth of bacterial pathogens. For example, the resident microbiota can modify the intestinal composition of carbohydrates and sugars present, which are essential for the growth of pathogens [98, 99]. Furthermore, many microbiota species such as Bifidobacteria and Lactobacillus are able to produce organic acids and SCFAs, which are detrimental to the growth of bacterial pathogens such as* S. Typhimurium* [100–102]. The microbiota, and in particular members of the* Clostridium* clusters IV and XIVa, now also known as* Ruminococcaceae *and* Lachnospiraceae,* produce the SCFA butyrate as a metabolite [103, 104]. Butyrate is an “antivirulence molecule” which acts as a diffusible signal to downregulate expression of the SPI-1 T3SS invasion genes in* Salmonella* [103, 105]. In contrast the SCFA formate induces the expression of the SPI-T3SS and invasion [106]. The SCFAs formate and acetate are largely located within the small intestine, whereas butyrate and propionate predominate in the colon [107].

### 3.5. Pathogen Clearance: The Microbiota and sIgA Have Complementary Protective Functions

It has been observed that following a* Salmonella* infection some individuals continue to shed* Salmonella* in stools once they have become asymptomatic [108]. These asymptomatic excretors are a major transmission risk. To investigate this phenomenon, Baumler and colleagues extended the coverage time of the streptomycin-mouse model to encompass later stages of infection when intestinal mucosal inflammation has begun to decline [109]. As discussed later in this review when the mammalian gastrointestinal tract becomes infected with* Salmonella*, there is an outgrowth of the pathogen at the expense of the resident microbiota, leading to a substantial reduction in its population size. Following an episode of such an acute infection and decreasing inflammation, pathogen clearance steadily reverses this process by reducing the luminal pathogen load and allowing the microbiota to regrow. This eventually leads to restoration of microbiota composition and numbers to steady-state levels. The mechanistic basis of pathogen clearance remains to be elucidated and conceivably may be multifactorial. For example, it is possible that the microbiota may generate inhibitory molecules or stimulate immunity. The gastrointestinal tract barrier function may also stabilize. An adaptive secretory IgA (sIgA) response is elicited during the later stages of infection and does not appear to play a significant role during this phase of clearance. However, if mice encounter this pathogen again, sIgA specific for* S. Typhimurium* LPS O-antigen appears to prevent inflammation. Hence, the microbiota and sIgA work in synchrony to protect against pathogens. This is evidently an important area of future research and undoubtedly will have important implications for other pathogens [110]. Nevertheless, host resistance to colonization can be manipulated by successful bacterial pathogens. There have been significant insights into our understanding of the ingenious counter-mechanisms employed by enteropathogens to bridge these lines of defence. These are discussed in later sections.

### 3.6. *Salmonella* Can Exploit the Hydrogen Produced by the Resident Microbiota to Jump-Start Initial Growth

In a normal healthy gastrointestinal tract, the resident microbiota generates hydrogen as a central chemical intermediate of metabolism. Wolf-Dietrich Hardt and colleagues have screened for essential genes required during infection in vivo using a transposon mutant bank [111]. Remarkably they identified* hyb* hydrogenase as playing a key role during the initial growth phases of* Salmonella* infection, and the findings were validated by a variety of methods including competitive infection experiments. Compelling evidence provided by the authors suggest that during these initial phases of infection,* Salmonella* can harvest or “steal” the hydrogen produced by the resident microbiota to “jump-start” and fuel their growth bloom [111].

## 4. *Salmonella* Fires Up Mucosal Inflammation in the Gastrointestinal Tract

Enteric pathogens, such as* Salmonella*, have devised strategies to unlock colonization resistance, compete with microbiota, and successfully infect the host. Vivid insights into the strategies deployed by* Salmonella* to compete with the resident microbiota have been provided by landmark studies from the laboratories of Andreas Baumler and Wolf-Dietrich Hardt, and colleagues [112, 113]. These studies and their remarkable findings are discussed in the sections below.

### 4.1. Inflammation Unlocks Colonization Resistance

**Figure 2:**
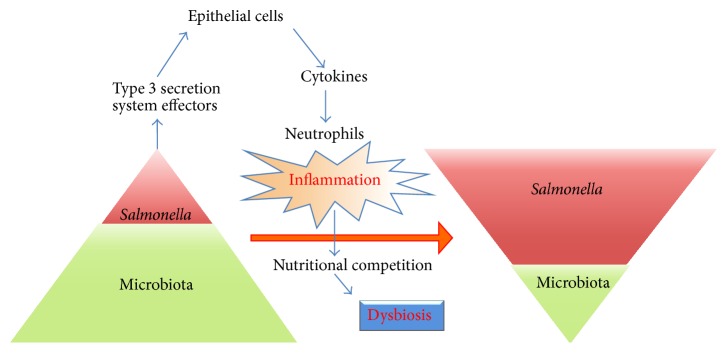
*Salmonella* are able to elicit host inflammation to ultimately inverse the intestinal bacterial population pyramid and become the dominant species. The intestinal microbiota exists in a delicate balance within the healthy host. An invading enteropathogen, such as* S*.* Typhimurium*, disturbs this balance by its interactions with the host and resident microbiota.* S*.* Typhimurium* type 3 secretion effector proteins trigger the release of cytokines and inflammation. This leads to a growth burst of* S*.* Typhimurium*, at the expense of the resident microbiota resulting in inversion of the bacterial population pyramid and dysbiosis.

In the mouse colitis model Wolf-Dietrich Hardt and colleagues observed that the potent inflammatory responses elicited by wild-type* S. Typhimurium* provided the pathogen with a major competitive growth advantage. This leads to a reduction in the population size of the microbiota and its composition and hence dysbiosis (Figure 2) [114, 115]. Surprisingly an avirulent* S. Typhimurium invG*,* sseD* mutant with disruptions in the SPI-1 and SPI-2 T3S systems failed to induce an inflammatory response and the mutant was unable to compete with the microbiota. However, this mutant phenotype could be rescued by inducing inflammation either by coinfection with the wild-type strain or using a transgenic strain of mice IL10^−/−^, VILLIN-HA^CL4-CD8^, designed to mimic the inflammatory state in irritable bowel disease. Hence, inflammation is key to surmounting colonization resistance. Thus, inflammation can in some instances benefit the pathogen at the expense of the host and microbiota [115].

### 4.2. Mucosal Inflammation Generates Exclusive Terminal Electron Acceptors for Respiration

*S. Typhimurium* is able to infect intestinal epithelial cells and survive within the hostile environment of professional macrophages. This is possible because* Salmonella* harbor the SPI-1 and SPI-2 T3SS that enable them to invade and survive within these cell-types. The effector proteins from these secretion systems, for example, SopE, are translocated into host cells to elicit the production of proinflammatory cytokines and potent inflammatory responses [116]. The inflammatory responses are amplified by T-cells located in the intestinal mucosa and release important cytokines.

**Figure 3:**
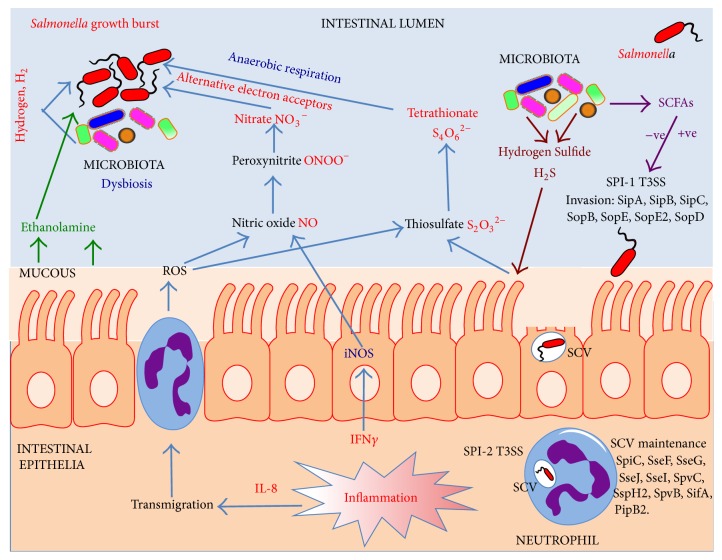
*Salmonella* use a variety of elegant strategies to compete with the intestinal microbiota. During the early stages of intestinal infection,* Salmonella* are able to scavenge the hydrogen produced by the microbiota to fuel initial growth. Microbiota produced short chain fatty acids (SCFAs) can modulate the expression of* Salmonella* SPI-1 invasion genes both positively (formate) and negatively (butyrate). Using the SPI-1 and SPI-2 type 3 secretion systems,* S*.* Typhimurium* are not only able to promote invasion and survival within host cells but also able to strategically elicit a host inflammatory response, which ultimately benefits the pathogen. The microbiota produces hydrogen sulfide, which normally becomes detoxified by host cells to thiosulfate. An inflammatory response leads to the transmigration of neutrophils into the intestinal lumen and the subsequent release of reactive oxygen species (ROS). When thiosulfate is exposed to ROS it is oxidized to tetrathionate, which can be exclusively used by* S*.* Typhimurium* as an alternative electron acceptor.* S*.* Typhimurium* can now utilize alternative carbon sources from the host, such as ethanolamine, using tetrathionate in anaerobic respiration. The inflammatory response results in the release of cytokines such as interferon-gamma (IFN*γ*). This results in the induction of expression of inducible nitric oxide synthase (iNOS), which generates nitric oxide. Upon exposure to superoxide free radicals nitric oxide is generated, and when exposed to ROS nitric oxide is converted to peroxynitrite and then nitrate. Nitrate can be used exclusively by* S*.* Typhimurium*, as an alternative electron acceptor during anaerobic respiration. This leads to an enormous growth burst in the pathogen leading to dysbiosis. Nitrate is thermodynamically the preferred electron acceptor over tetrathionate.

It has been observed that during* S*.* Typhimurium* infections, neutrophils transmigrate in to the intestinal lumen and release reactive oxygen species (ROS) to destroy invading pathogens [117]. The strictly anaerobic Bacteriodetes are dependent on complex polysaccharides and amino acids for fermentation and energy generation [96]. An end product of this fermentation is highly toxic hydrogen sulfide (H_2_S). This molecule is detoxified by colonic epithelial cells to produce thiosulfate (S_2_O_3_^−2^). However, when thiosulfate becomes exposed to ROS released by neutrophils, it is converted to tetrathionate (S_4_O_6_^−2^; Figure 3) [118]. Andreas Baumler and colleagues have vividly demonstrated that the majority of the microbiota species are unable to biochemically use tetrathionate, but remarkably* S. Typhimurium* is able to exploit tetrathionate as a terminal electron acceptor in anaerobic respiration (Figure 3) [118].

Several decades ago a multidrug resistance* S. Typhimurium* emerged which contained the SopE virulence gene within a prophage. SopE encodes for a SPI-1 T3S effector protein, which stimulates immune signaling pathways and also inflammation of the gastrointestinal tract [116, 119]. The prophage also enhanced the fitness of the strain by unknown mechanisms, and so its role in the mouse colitis model was examined. Baumler and colleagues found that SopE triggers the host to inadvertently generate nitrate, a thermodynamically more efficient terminal electron acceptor than tetrathionate [58, 120]. In the mouse colitis model they observed that there was an increase in mucosal inducible nitric oxide synthetase (iNOS) production following an* S. Typhimurium* infection. iNOS generates NO which in the presence of ROS forms peroxynitrite (ONOO^−^). Subsequently, peroxynitrite isomerizes to nitrate (NO_3_^−^), a terminal electron acceptor that can promote the luminal growth of* S. Typhimurium* by anaerobic respiration (Figure 3). This nitrate respiration-dependent growth advantage was reduced in iNOS-deficient mice [58]. Interestingly, nitrate also suppresses the expression of bacterial genes involved in the utilization of tetrathionate. This hierarchical control ensures the most energetically efficient electron acceptor is used in the competitive environment of the large intestine. Nitrate thus fuels growth blooms of* S. Typhimurium* in the inflamed intestine [58, 120].

Thus, the utilization of nitrate or tetrathionate as terminal electron acceptors in respiration is a far more efficient process for energy generation than fermentation. This provides a potentially huge competitive advantage to* S. Typhimurium* over the resident microbiota and allows the pathogen to access new carbon sources.

### 4.3. Terminal Electron Acceptors Enable* Salmonella* to Utilize Unique Substrates for Fermentative Growth Leading to Blooms

The gastrointestinal tractprovides an environment rich in diverse nutrients but the substrates able to support fermentative growth in the anaerobic environment are limited. The situation is exacerbated during inflammatory diarrhea where the rich contents of the gut are rapidly flushed out of the body and no longer available. In the inflamed gastrointestinal tract nutrients may become limited to mucous-derived carbohydrates and the contents of damaged intestinal epithelial cells such as the membrane lipid, phosphatidylcholine. In fact phosphatidylcholine can be readily metabolized to ethanolamine in mammalian intestines. Indeed in calve intestines ethanolamine is readily observed at a concentration of approximately 2 mM [121]. Baumler and colleagues have investigated the ability of* S. Typhimurium* to utilize ethanolamine [122]. They observed in vitro that ethanolamine could support the growth of* S. Typhimurium* only in the presence of tetrathionate. To investigate this in vivo, the mouse colitis model was used where mice were treated with streptomycin to disrupt the normal microbiota and then orally infected with a panel of* S. Typhimurium* mutants including those unable to induce inflammation, for example, SPI-1 and SPI-2 T3S mutants, ethanolamine utilization (*eutC*), and tetrathionate reductase (*ttrA*). Through a series of experiments they concluded that in the inflamed gastrointestinal tract,* S. Typhimurium* is able to use tetrathionate respiration to consume ethanolamine as a nutrient [122]. Ethanolamine cannot be used by the majority of the bacteria in the gut and thus confers* S. Typhimurium* with a huge growth advantage, which it indeed exploits. The resulting growth bloom in the gastrointestinal tract enhances transmission. Thus,* Salmonella* has used an ingenious mechanism to trigger inflammation to its benefit but detrimental to the host and competing microbiota.

### 4.4. Chemotaxis and Flagella-Mediated Motility Enables* Salmonella* to Identify and Swim to Nutritionally Beneficial Niches

Recent findings using a panel of mutant strains suggest that chemotaxis is important for growth of* Salmonella* in the inflamed intestinal tract and is dependent on flagella-mediated motility. These attributes may enable* S. Typhimurium* to migrate to suitable nutritional environments to maximize growth potential. Using chemotaxis,* S. Typhimurium* senses sugars such as galactose, which is present in high concentrations within the cecal mucosa, and these potential substrates may provide a chemotactic signal. However, the signals are also present in the noninflamed gastrointestinal tract, so what is the nature of the in vivo signal in the inflamed gut? To answer this question, Rivera-Chavez et al constructed a series of mutations in the methyl accepting chemotaxis proteins and the strains examined in the mouse colitis model [123]. The methyl accepting chemotaxis proteins Aer and Tsr were observed to enhance fitness by chemotaxis towards electron acceptors tetrathionate or nitrate, respectively, when these were required to provide a growth advantage in vivo. Thus, the methyl accepting chemotaxis proteins clearly enable the pathogen to “taste” their way to a nutritionally favorable niche and facilitate growth blooms [123].

### 4.5. The Gastrointestinal Tract Provides an Environment Conducive to Horizontal Gene Transfer amongst Residents

The entire coding capacity of the microbiota is referred to as the gene pool or “microbiome.” The microbiome can become altered by a variety of factors that affect the abundance of bacterial species. As mentioned previously these factors range from dietary changes, the use of antibiotics, and disease states to the acquisition of new species including pathogens. The resident species can acquire subtle genetic changes by natural point mutations or by the “brute force” of horizontal gene transfer (HGT) [124]. The latter processes enable species to acquire entire blocks of genes, for example, those involved in metabolism, antibiotic resistance, or encoding new virulence factors. Thus, HGT enables the species to evolve at a rapid rate in contrast to the slow accumulation of random point mutations. HGT takes place most efficiently between closely related species, for example, between Enterobacteriaceae. Furthermore, there is strong genomic evidence to suggest that HGT has taken place within the intestinal microbiota [125]. For example, it has been suggested that resistance to bile conferred by a bile salt hydrolase (*bsh*) present in* Bacteroides*,* Bifidobacterium*,* Clostridium*,* Lactobacillus*, and* Enterococcus* may have been acquired by HGT [126]. Under appropriate conditions HGT can also be observed between Enterobacteriaceae and Gram-positives and profoundly even across kingdoms [127–129]. Mechanistically, HGT can take place by transformation, transduction, and conjugation.

*DNA Transformation.* The uptake of released DNA from “donor” cells in the environment and its stable incorporation and expression within the recipient cells leads to DNA transformation. The ability of recipient strains to take up DNA and recombine it within their genomes is referred to as competence. Transformation in the gastrointestinal tract is expected to be infrequent due to the conditions, which do not favor the survival of free naked DNA. However, it has been suggested that stresses in the gastrointestinal tract may promote competence [130]. Indeed environmental cues such as the presence of chitin have been shown to induce the natural competence of* Vibrio cholerae* [124].

*Phage Transduction*. The DNA of host bacterial cells can be encapsulated by phages and transferred horizontally to recipient bacterial cells, in a process referred to as phage transduction. To promote their own survival within bacteria, phages have acquired additional genes not necessary for their lifecycle but provide a selective advantage for the host bacterium when the phage has integrated into the bacterial genome and is now a prophage. These fitness genes are known as “morons” and make the bacterium more competitive in the intestinal environment. Phages are physically resilient structures, which are expected to survive effectively in the milieu of the gastrointestinal tract. Transduction has clearly been an important mechanism for the transmission of genes as evidenced by genome sequencing where it has been uncovered that many virulence factors in enteropathogens are encoded by prophages. For example, in* S. Typhimurium* it has been observed that the T3S effector protein SopE is encoded by a prophage [131]. Strikingly there is new evidence to suggest that the acquisition of SopE by HGT stimulates the host to produce the electron acceptor nitrate to fuel a growth bloom of* S. Typhimurium* in the gastrointestinal tract [58]. Further in the Gram-positive* Enterococcus faecalis*, a facultative anaerobe and commensal of the human gastrointestinal tract, the acquisition of a composite prophage boosted the fitness of* E. faecalis* both in vitro and in vivo within the mammalian intestine [132]. These observations suggest that prophages associated with intestinal bacteria have a significant impact in shaping the bacterial communities in the gastrointestinal tract.

*Plasmid Conjugation.* Direct physical contact between donor and recipient bacteria are required for conjugative transfer of DNA. Conjugative plasmids are transferred to recipient cells through a conjugative pilus encoded by the plasmid. Not surprisingly conjugation efficiency increases with higher cell densities for the donor and recipient cells enabling more conjugative events to take place. As bacterial cell densities are very high within the gut, this niche will provide an environment perfect for promoting conjugative exchange of DNA. These exchanges are facilitated by the optimal growth conditions provided by an influx of nutrients within the gastrointestinal tract and a constant temperature of 37°C.

*Norepinephrine Can Modulate HGT.* The catecholamine norepinephrine is produced within the gastrointestinal tract, and it has been observed in vitro that norepinephrine, at physiological concentrations, promotes conjugative transfer of a large multidrug resistance plasmid from a clinical strain of* S. Typhimurium* to an* E. coli* recipient [133]. Furthermore, this effect appeared to be inhibited by the exposure of the cells to the *α*-adrenergic receptor antagonist phentolamine. The role of catecholamines in interkingdom signaling will be discussed in a later section.

*Inflammation Triggers Bacterial Growth Blooms Which Promotes HGT.* A major factor in promoting HGT is inflammation within the gastrointestinal tract leading to very high densities of Enterobacteriaceae [134]. Enterobacteriaceae are normally present in the gastrointestinal tract at low cell densities (<10^8^ cells/mL). Growth blooms in these bacteria can occur due to inflammation caused by invading pathogens such as* Salmonella*, as discussed already [115]. These blooms are fuelled by the increased availability of high-energy nutrients, which can be used by Enterobacteriaceae or the pathogens but not the resident microbiota. In some elegant studies by Hardt and colleagues it was demonstrated that when mice become infected with* S. Typhimurium*, the resulting inflammatory responses leads to growth blooms of the pathogen. Furthermore, the resident* E. coli* are also able to benefit from the products of these inflammatory responses. This results in a rapid elevation in enterobacterial numbers by several orders of magnitude leading to dysbiosis [134]. The increased density of the Enterobacteriaceae facilitates elevated physical contact, resulting in prolific conjugative rates from* S. Typhimurium* to* E. coli* of a conjugative plasmid encoding the bacteriocin colicin 1b. Thus, inflammation clearly promotes HGT between closely related bacteria.

Interestingly, Stecher and colleagues have recently demonstrated that within enterobacterial blooms, colicin Ib-mediated killing of competing commensal* E. coli* confers a distinct growth advantage to* S. Typhimurium* [135]. The genes encoding colicin Ib (*colIb*) and its receptor CirA were upregulated in* S. Typhimurium* and* E. coli, *respectively, during inflammation [135]. The mosaic genome of* Salmonella*, containing pathogenicity islands, prophages, transposons, and plasmids, provides evidence for significant levels of HGT during the evolution of this pathogen [11, 18, 136]. Furthermore, in a clinical setting, HGT in the inflamed gut may promote the spread of antibiotic resistance genes between the microbiota and invading pathogens and subsequently selected by antibiotic therapy.

### 4.6. Host Toll-Like Receptor Sensing of* Salmonella* Activates the Bacterial Virulence Machinery

The innate immune system plays a vital role in controlling infections once a pathogen has been detected. The system recognizes pathogen-associated molecular patterns (PAMPs) and sets in to play a series of signaling cascades designed to eliminate the pathogen and warn the adaptive immune system of infection [137–139]. PAMPs are recognized by a special family of proteins called toll-like receptors (TLRs). When triggered, TLRs recruit host cell adaptor proteins including MyD88 and TRIF, which activate signaling cascades to promote defense of the host. Each TLR recognizes a particular signature; for example, TLR-4 recognizes lipopolysaccharide from the outer membranes of Gram-negative bacteria, whereas TLR-5 detects bacterial flagellin [140]. TLRs are distributed on the surfaces of many cell-types including macrophages and mucosal epithelial cells.

As discussed earlier* Salmonella* reside and replicate within host cells such as macrophages by transforming the hostile environment of the phagosome into the* Salmonella*-containing vacuole. They survive these harsh conditions by turning on the expression of SPI-2 T3SS genes. The T3S effectors enable the pathogen to manipulate the environment of the host cell for its benefit. What are the signals, which turn on expression of the SPI-2 T3SS? Holden and colleagues demonstrated through some elegant studies that expression of SPI-2 T3S is triggered by acidic pH [73]. Using a panel of transgenic TLR knock-out mice, Arpaia et al. have shown that acidification of the phagosome is in fact activated by TLR signaling which rather than protecting the host actually now benefits the pathogen [139].

### 4.7. Extinguishing Inflammation in the Gastrointestinal Tract: Hypoarginemia Elevates Susceptibility to* Salmonella* Infection

The gastrointestinal tract is one of the most metabolically active tissues in humans and has high levels of protein synthesis and cell turnover. Regulation of the gut barrier function is crucial for preventing disease and maintaining good health. The amino acid L-arginine (L-Arg) is not only required for protein synthesis but also appears to be important as a regulator of intestinal function and homeostasis. Indeed the availability of extracellular arginine can impact upon immune defense [141]. This is because arginine is a substrate for inducible nitric oxide synthetase iNOS to generate nitric oxide. Nitric oxide is an effective antimicrobial agent produced by macrophages to combat pathogens but as discussed in this review, nitric oxide can also be exploited by* Salmonella* through its inflammatory response and used to generate nitrate, which can subsequently be used as a terminal electron acceptor (Figure 3) [58]. Recently it has been observed that malaria-patients develop L-arginine deficiency which reduces intestinal barrier function and makes the patients vulnerable to coinfection with* S. Typhimurium* [142]. These effects can be compensated by supplementing the diet of patients with arginine, which leads ultimately to improved intestinal barrier function and protection against infection by* S. Typhimurium* [143].

## 5. *Salmonella* Show Their Metal under Fire

To successfully compete against the resident microbiota within the inflamed gastrointestinal tract,* Salmonella* must acquire vital nutrients. Some of these nutrients include metals such as iron, zinc, copper and are essential for growth and proliferation [144, 145].

### 5.1. *Salmonella* Can Defend against the Effects of Antimicrobial Peptides and Sequester Precious Metals

Within the gastrointestinal tract, pathogens are under attack from host antimicrobial peptides such as lipocalin-2, which are secreted by the intestinal epithelial cells and protect against invading bacteria [146]. Intestinal infections with* S*.* Typhimurium* lead to an increase in IL-17 and IL-22 production, which in turn stimulate the intestinal cell production of lipocalin-2, which subsequently accumulates in the lumen. Iron is an essential metal required by bacteria including* S*.* Typhimurium*. To scavenge for iron, bacteria can secrete siderophores, such as enterochelin, which bind any available iron in the environment and are subsequently taken up by the bacterial cell. Lipocalin-2 is a 24 kDa glycoprotein which binds to bacterial siderophores, thus starving bacteria of essential iron and preventing growth [147]. However,* S. Typhimurium* are resistant to the effects of lipocalin-2 as they are able to synthesize an alternative iron binding protein salmochelin. The synthesis and uptake system for salmochelin are encoded by the* iroBCDE* and* iroN* genes [148–153]. Salmochelin is a glycosylated derivative of enterochelin, which does not bind lipocalin-2 and is thus resistant to its effects. This resistance provides* S. Typhimurium* with a significant growth advantage against competing bacteria in the inflamed intestine [146].

Bacterial pathogens also face a barrage of attack from neutrophils, which have migrated into the intestinal lumen. Approximately 40% of the cytoplasmic nutrient content of neutrophils is composed of a protein named calprotectin [117]. Calprotectin has potent antimicrobial activity against many bacterial pathogens including* E. coli *and* Listeria monocytogenes*, due to its ability to bind and sequester essential metals such as zinc and manganese. Raffatellu and colleagues observed that in the presence of* S. Typhimurim*, neutrophils are induced to release calprotectin [154]. However,* S*.* Typhimurium* is able to survive the effects of calpoprotectin by expressing a high affinity zinc transporter (ZnuABC). This transporter enables the pathogen to grab zinc and provides a growth advantage over the competing bacteria in the inflamed environment of the gastrointestinal tract.

### 5.2. Probiotic Bacteria Pump Iron to Raise Fitness and Outcompete* Salmonella*

Probiotics are commensal organisms that provide benefits to the host by direct interactions or by competition with pathogens as discussed above in colonization resistance. During an outbreak of shigellosis, a probiotic strain was isolated from a soldier who appeared resistant to an outbreak of diarrhea [155]. The probiotic strain was* Escherichia coli* Nissle 1917 (serotype O6:K5:H1). The* E. coli *Nissle strain colonizes the gastrointestinal tract efficiently and has been successfully used as a probiotic for treating intestinal disorders by unknown mechanisms [156]. As acquisition of nutrients such as iron are important for* S. Typhimurium*, the genome of* E. coli* Nissle was examined and revealed the presence of multiple iron uptake systems. These include salmochelin, the mixed-type siderophore yersiniabactin, the hydroxamate-type siderophore aerobactin, and the hemin uptake transporter ChuA [157]. Rafettellu and colleagues hypothesized that iron uptake mechanisms are vital for* E. coli* Nissle probiotic activity. Using the* S. Typhimurium* colitis mouse models the investigators observed that this nonpathogenic* E. coli* Nissle was able to outcompete and reduce the size of* S. Typhimurium* populations during mixed infections. However, when an iron uptake mutant of* E. coli* Nissle was examined it was found to colonize the intestinal tract but was now unable to dampen down the* S. Typhimurim* population size [157]. Using the mouse models to examine the impact in further detail, it was observed that iron plays an essential role in promoting the competitiveness of* E. coli* Nissle in a lipoclain-2 system [157]. The inoculation of* E. coli* Nissle resulted in a massive reduction in colonization of* S. Typhimurium* and a general reduction in gastrointestinal inflammation. This study has provided valuable insights in to the mode of action of probiotics, and these organisms could be further developed to provide important benefits to a variety of intestinal diseases.

## 6. Multidirectional Chemical Signaling within the Gastrointestinal Tract

**Figure 4:**
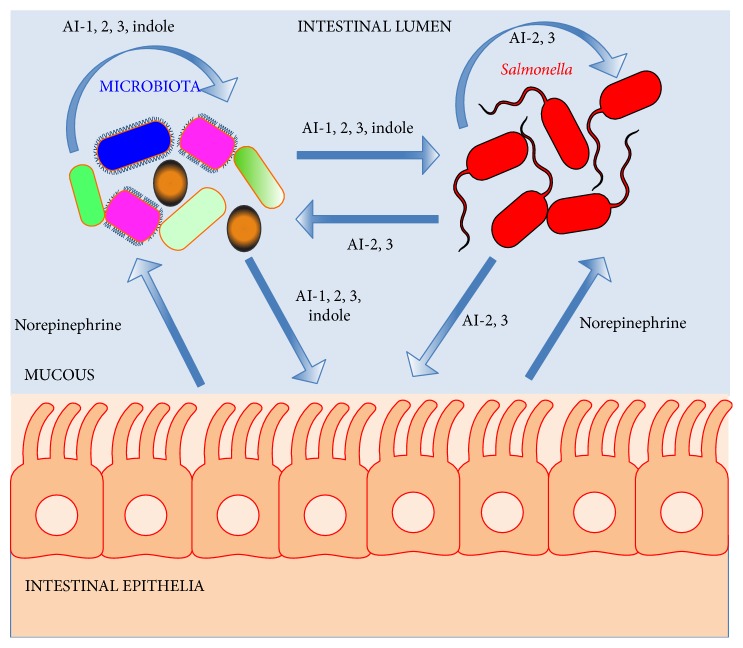
Chemical signaling between* Salmonella*, the intestinal microbiota, and the host. In the complex environment of the gastrointestinal tract there are opportunities for chemical signaling to take place between the microbiota, host cells, and the invading pathogen. The resident microbiota and* S. Typhimurium* may produce signaling molecules, which modulate the activities of the microbiota or pathogen. Examples of such signaling molecules include AI-1, AI-2, AI-3, and indole. Some of these signals may also modulate the activities of host cells such as AI-1, AI-3, and indole. The host can produce signaling molecules, which can also be detected by the microbiota and pathogens to modulate their activities. These host signals include catecholamine hormones such as norepinephrine.

The gastrointestinal tract contains a highly complex community of host cells, microbiota, together with invading pathogens. These diverse cellular communities provide remarkable opportunities for signaling at multiple levels between the resident microbiota and host cells, together with invading pathogens. Understanding the mechanistic basis of multidirectional chemical signaling will provide important insights into health and disease. These signaling processes may operate at the levels of quorum sensing or interkingdom communication (Figure 4).

### 6.1. Quorum Sensing with Bacterial Autoinducers

Bacteria synthesize small diffusible signal molecules to count and monitor their population density by a process termed “quorum sensing” [158, 159]. When a critical concentration of this signal molecule is reached, this information is relayed and used to coordinate gene expression within the population and modulate the expression of important phenotypes such as virulence, biofilm formation, and persistence [160]. The bacterially produced signaling molecules are referred to as autoinducers AIs and these molecules are generally very similar in related species [161]. There is now also increasing evidence to suggest that these AIs can also be sensed by species which do not produce the signals themselves, leading to interspecies signaling. These AIs could thus have a major impact on the composition and development of polymicrobial communities in natural settings such as in the environment or a host (Figure 4) [162, 163].

*Acyl-homoserine Lactone: An Enteric Signal.* Many Gram-negative bacteria, with the notable exceptions of* E. coli* and* Salmonella*, quorum sense with* N-*acyl-homoserine lactone signaling molecule [acyl-HSL] also known as autoinducer-1 [AI-1] [158]. LuxI synthesizes the QS signal molecule, which is subsequently detected by the cognate sensor and transcriptional regulator LuxR. Acyl-HSL can vary in their acyl side chain length from 4 to 18 carbons. Although neither* E. coli* nor* Salmonella* synthesize acyl-HSL, they do possess a LuxR orthologue known as SdiA. Consequently they can sense acyl-HSLs produced by other bacterial species in a process aptly termed “eavesdropping” [164]. These enterics can infect the intestinal tracts of humans and animals and it maybe that* Salmonella* and* E. coli* are able to detect acyl-HSLs produced by members of the intestinal microbiota and use these signals as an environmental cue to regulate gene expression. Barring physical degradation of the acyl-HSLs, there appears to be no chemical or biological evidence to support this notion. However, Ahmer and colleagues used a clever genetic screen, a recombination-based in vivo expression technology (RIVET) reporter system, in which SdiA dependent detection of acyl-HSLs would result in a permanent deletion of a tetracycline resistance gene during the passage of* Salmonella *through a host [164–167]. The* Salmonella *infection reporter screen did not detect any acyl-HSL during infections of a number of animals ranging from mice to chickens to cows. However, the reporter was found to become active in turtles and also in mice, which had been infected with the pathogen* Yersinia enterocolitica *[165]. Thus, it appears that the intestinal microbiota does not appear to produce detectable levels of acyl-HSLs, but some intestinal pathogens can generate acyl-HSLs enabling* S. Typhimurium* to eavesdrop on these quorum sensing pathogens when coinfecting the host. The competitive advantages of acyl-HSL eavesdropping for* Salmonella* remain to be elucidated.

*The “Universal” Signal Autoinducer 2.* The majority of both Gram-negative and Gram-positive bacteria possess the enzyme LuxS that is an AI-2 synthase or S-ribosyl homocysteine lyase, including members of the intestinal microbiota such as* Bacteroides* species. AI-2 is believed to be a QS signaling molecule, which has been referred to as a universal signaling molecule due to the widespread distribution of* luxS *[161]. In the well-studied* Vibrio harveyi* system, AI-2 is a furanosyl borate diester. In contrast to* S. Typhimurium*, AI-2 has the identity of (2R,4S)-2-methyl-2,3,3,4-tetrahydroxytetrahydrofuran (*R*-THMF) and lacks boron [168]. The role of AI-2 as a true QS signaling molecule remains to be unequivocally demonstrated in most cases [163, 169, 170]. In bacterial species studied to date AI-2/*luxS* appears to regulate expression of genes involved in virulence, biofilm formation, motility, and carbohydrate metabolism. In* S*.* Typhimurium*, AI-2 affects the expression of the AI-2 uptake system the Lsr operon [171–173]. However, LuxS plays an important metabolic role in the activated methyl cycle, and its role in metabolism could have an impact on the observed phenotypes. This is supported by the following observations. In* S. Typhimurium* it has been demonstrated that a* luxS* gene deletion mutant modulates flagellar phase variation independent of AI-2 leading to expression of phase-1 flagellin subunits [174, 175]. Complementation studies revealed this phenotype appeared to be dependent on the small RNA* micA* located immediately upstream to the* luxS *coding sequence rather than the* luxS* gene product itself [174, 176]. The same observation is true for* Salmonella* biofilm formation [175]. Furthermore, in* Streptococcus sanguinis,* complementation of the active methyl cycle with the* S-*adenosylhomocysteine hydrolase (SahH) gene restores biofilm formation independently of AI-2 and* luxS* [177]. The picture is also complicated as the* luxS* deletion mutant also has pleiotropic effects on* S*.* Typhimurium* gene expression [178]. As a consequence of the pleiotropic effects on bacterial metabolism, AI-2 has also been indirectly implicated in the production of another autoinducer (AI-3) in* E. coli* and also* Salmonella* [179, 180].

It has recently been demonstrated that* Bacteroides* species can produce AI-2 like molecules [181], and heterologous expression of* Bacteroides luxS* orthologues can complement AI-2 production in* E. coli* [182]. As members of the intestinal microbiota produce AI-2, it is not inconceivable that members of these dense communities exploit LuxS-based signaling to modulate gene expression and community phenotypes.

*Commensal Bacterial Indole Signals Diminishes the Pathogenicity of Salmonella.* There is increasing evidence to suggest that indole signaling is used by bacteria within the gastrointestinal tract for communication [183]. In commensal* E. coli* the environmental conditions of the intestine induce the expression of tryptophanase (*tnaA*), the enzyme that generates indole. Indole concentrations in the mammalian intestine can vary from 250 *μ*M to 1 mM [184].

Indole is produced by* E. coli *in stationary phase cells and appears to regulate biofilm formation, acid resistance, and the locus for enterocyte effacement in pathogenic* E. coli* [185]. Indole also elevates the expression of multidrug exporters and has an impact on population based antibiotic resistance in* E. coli*. Indole signaling clearly affects membrane and oxidative stress response. Following on from this it has been demonstrated that indole induces the formation of persisters, where a fraction of the isogenic bacteria in a population “tolerate” antibiotics. Persister formation was monitored by Collins and colleagues by the novel use of microfluidics, combined with fluorescence microscopy and DNA microarray screens [186]. They identified stress response pathways, including OxyR and phage stress response (Psp), which were essential for persister formation. Thus,* E. coli* can use a “bet-hedging” strategy when nutrients are limited by producing a heterogeneous population of bacteria to improve their chances of survival [186].

Curiously* S*.* Typhimurium* is unable to produce indole as it does not have the tryptophanase enzyme. However, indole has been observed to induce the expression of the* S. Typhimurium acrAB-tolC* multidrug efflux system and this phenotype is dependent upon the regulator RamA. Intriguingly using DNA microarrays the authors observed that indole downregulated the expression of the SPI-1 T3SS genes and also genes involved in motility [185]. These changes were directly manifested phenotypically by a reduction in invasiveness and motility. These phenotypes as discussed elsewhere in this review are clearly important for eliciting an inflammatory response in the host and benefits* Salmonella* competitively at a nutritional level over competing microbiota, including commensal* E. coli,* leading to growth blooms. Thus, this may be a mechanism designed by members of the microbiota to dampen the competitiveness of* S. Typhimurium* and reduce the growth blooms. This suggests that indole could potentially be used therapeutically to control infections against bacterial pathogens such as* S. Typhimurium*.

*Intestinal Cells Can Intercept Bacterially Produced Indole to Fortify Host Defense Barriers. *From the previous sections there is convincing evidence to suggest interkingdom communication can take place by bacteria eavesdropping on host signaling molecules such as hormones (Figure 4). Bacteria also exploit indole as a signaling molecule as discussed. The physiological impact of bacterially produced indole on host intestinal cells was investigated by Jayaraman and colleagues [187]. They observed changes in gene expression in the human enterocyte HCT-8 cell line when exposed to physiological concentrations of indole using DNA microarrays and phenotypic screens. They observed that indole increased expression of anti-inflammatory genes and the downregulation of some proinflammatory genes. Furthermore, genes involved in enhancing the mucosal barrier such as actin-cytoskeleton and tight-junction proteins were elevated, with increases in mucin gene expression and production. This led to an increase in the transepithelial cell resistance of the HCT-8 cells suggesting the mucosal barrier had been fortified [187]. This reduces the ability for enteric pathogens to traverse the intestinal epithelial cell barrier by increasing its integrity and resistance to invasion. The findings of this research also suggest that indole could be used to treat inflammatory intestinal bowel disorders such as Crohn's disease by enhancing the epithelial cell barrier and dampening inflammation.

### 6.2. Interkingdom Signaling within the Gastrointestinal Tract

Catecholamines such epinephrine and norepinephrine are key players in mediating acute host stress. Half of the norepinephrine in the human body is produced by the neuroendocrine system within the gastrointestinal tract. Through the original investigations by Lyte and colleagues it has now become established that bacteria can sense and respond to host produced signaling molecules such as norepinephrine and epinephrine [188, 189]. These findings have given birth to new field, aptly coined by Lyte as “microbial endocrinology” (Figure 4) [190].

*Microbiota Play a Pivotal Role in the Production of Catecholamines. *Catecholamines are produced by the neuroendocrine system within the gastrointestinal tract and it was believed the resident commensals played no role in this process. To determine whether the intestinal microbiota had an impact on catecholamine production, Asano and colleagues have overcome some major technical challenges and determined the physical levels of catecholamines norepinephrine and dopamine using HPLC [191]. Using pathogen-free mice, germ-free mice, and gnotobiotic mice, the authors determined the catecholamine levels in the gastrointestinal tract of these mice. Their studies revealed that pathogen-free mice had elevated levels of catecholamines when compared to germ-free mice, which had no intestinal microbiota. However, the introduction of either: specific pathogen-free mice fecal bacteria,* E. coli*, or* Clostridium *species into germ-free mice resulted in a remarkable increase in the levels of biologically active dopamine and norepinephrine. Furthermore, the authors went on to observe that an* E. coli* mutant unable to produce *β*-glucuronidase was no longer able to raise the levels of free biologically active catecholamines, thus suggesting the bacterial enzyme plays a key role in the hormone maturation process [191]. This powerful comparative approach has vividly revealed that the resident gut microbiota and the bacterial enzyme *β*-glucuronidase have pivotal roles in the production of biologically active catecholamines in the gastrointestinal tract of the host.

*Catecholamines in the Gastrointestinal Tract Regulate the Virulence of Salmonella.* The gastrointestinal pathogen enterohemorrhagic* Escherichia coli* (EHEC) harbors a pathogenicity island named the locus of enterocyte effacement (*LEE*). A relatively new bacterial autoinducer has been reported, AI-3, which stimulates the expression of the* LEE* virulence operon in EHEC colitis. The sensing of AI-3 and signaling takes place through the two-component signal transduction systems QseBC and QseEF. These two-component systems regulate virulence by modulating the expression of the* LEE* locus [192, 193]. Astonishingly the catecholamines epinephrine and norepinephrine can imitate the same biological effects of AI-3 [192, 193]. Furthermore, *α*- and *β*-adrenergic receptor antagonists can block the signaling.

These hormones also have an impact on the virulence of* Salmonella* [163, 174, 194, 195]. Lipopolysaccharide is an important molecule in the outer membranes of Gram-negative bacteria and is subject to modification to help adapt the bacterium to survive in different environments. For example, the structure of LPS can be modified by the* pmr* operon in* Salmonella* to adapt to different environments; for example, in the gastrointestinal tract Paneth cells are an important source of antimicrobial peptides. The* pmr* operon confers resistance to antimicrobial peptides such as polymyxin B. If* Salmonella* are exposed to epinephrine and subsequently challenged with polymyxin B, they become more sensitive to the effects of this cationic antimicrobial peptide [196]. However, the effect is fully reversible with the addition of propranolol, a *β*-adrenergic blocker [196]. The phenotype was independent of QseC, and through genetic screens the BasSR two-component signal transduction system was identified as being essential for this observation. The LPS modifying enzymes PmrF and PagL are down-regulated by epinephrine leading to an altered LPS chemotype. These modifications in the structural configuration of LPS can increase the sensitivity of the pathogen to antimicrobial peptides, and also subdue the host inflammatory responses as the modified LPS now reduces activation of the TLR-4 receptors [197].

The exclusively human pathogen* S. *Typhi can sense and respond to catecholamine hormones and release the toxin HlyE in outer membrane vesicles [198]. The signaling cascades involve the two-component system CpxAR, with increased expression of the sRNA* micA* and the RNA chaperone Hfq. This complex is believed to block the translation of the* ompA* mRNA leading to reduced amounts of OmpA in the outer membrane. This facilitates the release of outer membrane vesicles containing HlyE [163, 194, 198]. From the above examples, adrenergic signaling is clearly a double-edged sword, which can provide benefits to the pathogen but also on occasions to the host.

*Fucose Sensing and Intestinal Colonization. B. thetaiotaomicron* is believed to possess fucosidases, which cleave fucose from glycans such as mucin [199, 200]. Very recently it has been reported that fucose can be sensed by the gastrointestinal pathogen EHEC to regulate the expression of virulence genes located within the* LEE* locus [201]. Expression of genes in this locus is activated by the bacterial signal AI-3 and host catecholamines as described above. Sensing of fucose is mediated by a two-component system FusKR located on the pathogenicity island OI-20 and appears to downregulate expression of the* LEE* locus [201]. Furthermore, QseBC and QseEF repress the expression of FusKR. These findings suggest that EHEC is able to sense fucose produced by the intestinal mucosa and subsequently modulate the expression of virulence genes [201]. The rationale behind this reciprocal regulation is puzzling, as the catecholamines and AI-3 signal would be expected to be in very similar locations as the fucose signal. Clearly further research is required to definitively answer this question and also whether other enteric pathogens such as* S. Typhimurium* are able to sense and respond to fucose to modulate virulence gene expression.

## 7. Concluding Remarks and Future Perspectives

*Salmonella* possess an armory of virulence factors to enable efficacious infection of their hosts. Many of these virulence factors protect the pathogen against host defenses and enable* Salmonella* to invade and colonize mammalian tissues. Central to these processes are the SPI-1 and SPI-2 T3SSs, which have been acquired by ancestor strains through horizontal gene transfer. There are a wealth of interactions between* Salmonella*, the microbiota, and the gastrointestinal tract. In contrast to* S*. Typhi, it is believed that* S. Typhimurium* deliberately engages the host inflammatory response through the SPI-1 and SPI-2 T3SSs. The inflamed intestine provides* S. Typhimurium* with competitive growth advantages over the resident microbiota, by enabling the generation of unique electron acceptors and the subsequent utilization of new substrates by the pathogen.* S. Typhimurium* can thus overcome “nutritional immunity” by strategically modifying its immediate environment enabling them to efficaciously outcompete the resident microbiota. It is essential to determine the contents of the inflamed intestine by metabolomics to facilitate the identification of potential substrates and understand how* Salmonella* manipulate the nutritional environment of the gastrointestinal tract. Anaerobic respiration could also provide a therapeutic target to control infections, and this needs to be explored.

The intestine provides a very rich and dense ecosystem enabling complex signaling between the host gastrointestinal cells, the resident microbiota, and invading bacterial pathogens. Bacteria generate a plethora of signaling molecules and through their intimate interactions with mammalian hosts have acquired the abilities to intercept host signals. Metabolomic approaches to comprehensively analyze intestinal contents may facilitate the identification of such signaling molecules. Understanding these elaborate signaling cascades will be central to gaining a full understanding of the processes required for maintaining good health and preventing disease.

The present research at understanding the interactions between the host cells, microbiota, and bacterial pathogens has been limited to model organisms. These models have proved very valuable but have limitations. Future research should aim to examine these interactions directly within the natural hosts for* Salmonella*, from livestock to humans. The rapid technological advances taking place in rRNA sequencing as well as whole-genome sequencing will facilitate these systems biology based approaches. Investigators are now shifting their objectives from collecting a descriptive analysis of the microbiota community composition and their associations with health and disease to delivering more fundamental mechanistic insights into the dynamic interactions between the host, resident microbiota, and invading pathogens.
